# Mitotic error correction and the spindle assembly checkpoint: a tension-filled relationship

**DOI:** 10.1080/15384101.2026.2653527

**Published:** 2026-04-03

**Authors:** Richard Pleuger, Stefan Westermann

**Affiliations:** Department of Molecular Genetics I, Faculty of Biology, Center of Medical Biotechnology, University of Duisburg-Essen, Essen, Germany

**Keywords:** Mitosis, kinetochore, tension sensing, signal transduction, protein phosphorylation

## Abstract

The fidelity of mitotic chromosome segregation relies on kinetochores detecting sister chromatid bi-orientation to control error correction (EC) and the spindle assembly checkpoint (SAC). The kinetochore-microtubule attachment state needs to be decoded, the signal processed, and transduced, resulting in either stabilization or destabilization of the attachment. Although many crucial players of this process have been identified, the molecular mechanisms underlying signal integration remain an open question. Focusing on the model system *Saccharomyces cerevisiae*, we explore the interdependent contributions of the conserved protein kinases Mps1 and Ipl1^Aurora B^, crucial regulators of mitotic chromosome segregation. We discuss how bi-orientation reorganizes the kinetochore attachment site and we present a perspective on how these structural changes can alter kinase localization and activity.

## 1. Introduction

**Figure 1.**
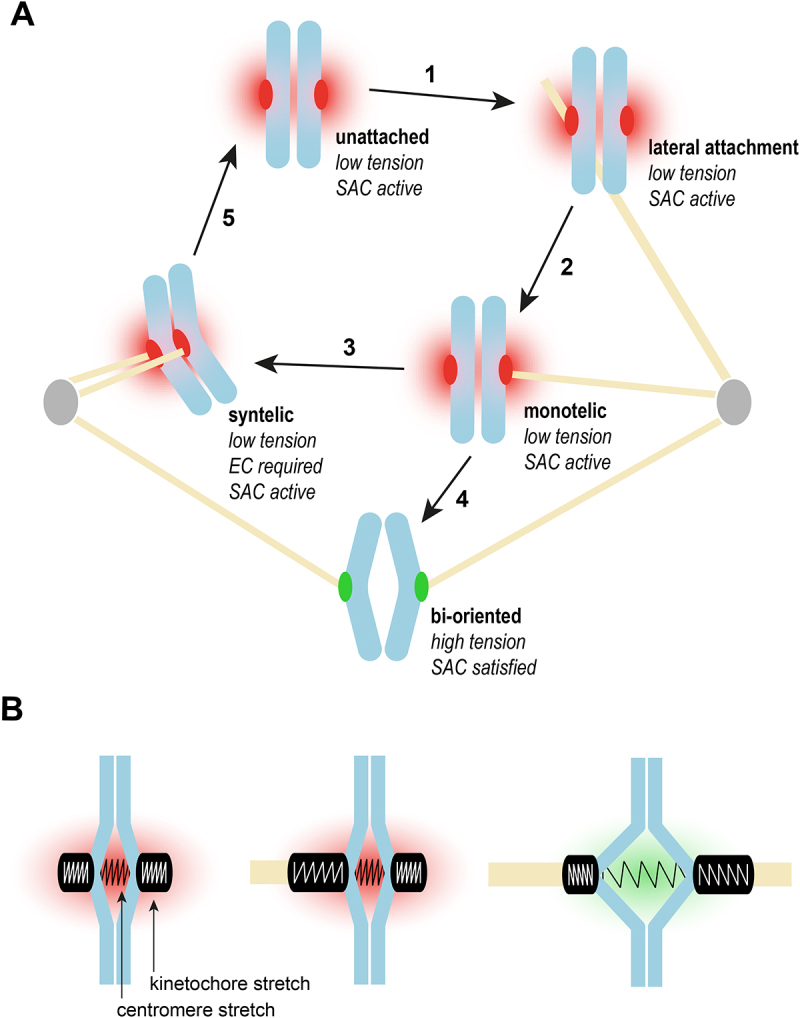
The maturation of kinetochore-microtubule attachments A. Unattached kinetochores initially form lateral attachments (1). Upon lateral to end-on conversion (2), the chromosome arrives in a mono-oriented attachment state. The kinetochore on the other side can attach to a microtubule emerging from the same spindle Pole (3) or the opposing one (4). Consequently, the chromosome is rendered in a syntelic or a bi-oriented attachment state, respectively. The lack of tension in the syntelic attachment is detected and error correction induces the release of the microtubules (5). B. Schematic illustration of stretching of the centromere/kinetochore framework. While unattached, both centromere and kinetochores are in a relaxed state. Once a microtubule attaches in an end-on configuration, the attached kinetochore can display kinetochore stretching. The centromere is stretched in a bi-oriented attachment configuration, while the corresponding kinetochores can display either a stretched or an relaxed morphology.

Faithful chromosome segregation during cell division requires the mitotic spindle, the specific organization of microtubules built during mitosis, to equally segregate the two sister chromatids into the two daughter cells. In order to accomplish this, all chromosomes need to achieve bi-orientation, which describes a state where the two sister chromatids of one replicated chromosome are connected to microtubules arising from the opposing poles of the spindle (Figure 1A). Given that attachments between microtubules and chromosomes are thought to form stochastically (albeit with a bias toward bi-orientation [1,2]), other, non-favorable, attachment states occur: monotelic attachments (only one chromatid is attached) and syntelic attachments (both chromatids are attached to the same spindle pole), and – in systems where one chromatid attaches to multiple microtubules – merotelic attachments (one chromatid is attached to both spindle poles). The cell needs to detect these erroneous attachment states and induce correction mechanisms, a process known as error correction (EC). Further, the cell needs to prevent the metaphase to anaphase transition as long as even a single chromosome has not achieved bi-orientation. This cell cycle progression checkpoint is known as the spindle assembly checkpoint (SAC). Conductors of these processes are multi-protein complexes called kinetochores. Kinetochores are the anchor points between chromosomes and the mitotic spindle. They form load bearing end-on connections with the plus-tip of a microtubule, as well as lateral interactions with the microtubule lattice. Kinetochores also serve as a recruitment platform for the EC machinery and signaling hub for SAC effectors. Given the tight functional connection between EC and the SAC, researchers have spent the last decades dissecting their mechanistic relationship.

Here, we present an overview of the interconnected signaling networks crucial for mitosis with a focus on the model organism budding yeast (*Saccharomyces cerevisiae*). Taking a look at both historical and recent observations, we discuss the interdependence of two conserved protein kinases, Mps1 and Ipl1, the main players in the SAC and EC, and develop a perspective on how their activity is connected to the attachment state of a given kinetochore.

Given the emphasis on work in yeast, in the following sections proteins are predominantly named by their budding yeast names. If the concept is more broadly applicable, the human homologue is added to the protein name in the superscript. If the information comes from a specific experiments, the model organism is mentioned, and the proteins are named according to their host organism.

## 2. The concept: sensing chromosome bi-orientation

The mitotic checkpoint reads out the kinetochore’s attachment state, integrates the information, and makes a decision on whether the cell proceeds into anaphase. Understanding the signal transduction underlying mitotic progression requires the identification of the biophysical parameter that encodes the more abstract concept of bi-orientation. To this end, two main aspects are discussed: the physical attachment built between microtubules and kinetochores and the generation of tension resulting from pulling forces of the microtubules on sister chromatids and kinetochores.

Early experiments revealed that the SAC can be satisfied by applying tension-generating forces on chromosomes [3,4]. Kinetochore microtubule attachments are stabilized if tension is applied using micromanipulation techniques, while those lacking tension are destabilized. Under physiological conditions, forces are generated by the dynamic instability of microtubules and exerted on kinetochores to induce chromosome movement [5,6]. Differentiating the individual contributions of attachment and tension to mitotic signaling under physiological conditions is severely complicated from their interdependencies: tension requires microtubule attachment, stable microtubule attachment requires tension. To sense bi-orientation, tension provides another advantageous property: it requires the presence of opposing force vectors. In the mitotic spindle, these would be generated by load-bearing microtubules originating from opposing spindle poles, i.e., a bi-oriented attachment state. Neither a monotelic nor a syntelic attachment would provide this as the attached microtubules would exert force only in one direction. These connections require detachment by EC mechanisms (Figure 1A).

Although this model emphasizing spindle tension is often applied to understand mitotic fidelity, it does not sufficiently account for the question of which structural component is under tension. One can differentiate between tension that occurs along the centromeric chromatin (inter-kinetochore tension, also referred to as centromere stretch), and tension within one kinetochore (intra-kinetochore tension, also referred to as kinetochore stretch) (Figure 1B). Experimentally, inter-kinetochore tension is assessed by analyzing the distance between kinetochores on sister chromatids [7,8], whereas intra-kinetochore tension leads to increased distance between subunits of the inner kinetochore relative to the outer kinetochore [9–12]. It is important to note that the two modes of stretching are uncoupled, implying that the molecular mechanisms of mechanical transformation are distinct between the two [12,13].

It is not fully clear, how the forces stretching kinetochores are generated. A lateral attachment would not generate significant forces without motor proteins. An end-on attachment between kinetochores and microtubules is therefore required and it can occur in both mono- and bi-oriented kinetochores. The forces that generate intra-kinetochore tension can be generated within the framework of a single kinetochore and do not require the opposing forces from a bi-oriented attachment. This can be inferred from two observations: (a) two sister kinetochores stretch independently [13]; (b) attached kinetochores show stretching under mono-polar spindle conditions as well [14]. One model proposes that the forces driving kinetochore stretching are generated by an end-on attached microtubule that polymerizes against the inner kinetochore [14].

Another important question is which architectural elements are affected in the stretching of kinetochores. Kinetochores are built in a hierarchical manner from inner to outer kinetochore [15,16]. The inner kinetochore, known as constitutive centromere associated network (CCAN), forms tight connections with centromeric chromatin. The CCAN adapts an predominantly globular shape associated with a nucleosome that contains the centromere-specifying histone H3 orthologue Cse4^CenpA^ [17,18]. The outer kinetochore, also referred to as Knl1 complex : Mis12 complex : Ndc80 complex (KMN) network, is responsible for the interaction with microtubules and contains crucial elements of the SAC signaling cascade and the error correction machinery. In budding yeast, there is the additional Dam1 complex. It can form rings around microtubules and is required for stable end-on kinetochore attachments [19,20]. The two structural elements, inner and outer kinetochore, are connected though the proteins Mif2^CenpC^, Ame1^CenpU^ and Cnn1^CenpT^ [21,22]. Since Mif2, Ame1, and Cnn1 all contain long intrinsically disordered regions (IDRs), it is plausible that intra-kinetochore tension stretches these flexible connectors, increasing the distance between inner and outer kinetochore modules. Indeed, bypassing CenpT’s flexible linker domain by artificial dimerization of its N-terminal and C-terminal domain compromises the observed kinetochore stretch [14]. In this experiment, the artificial limitation of kinetochore stretching results in checkpoint silencing delays.

Structured components can contribute to the increased distances within the kinetochore as well. The Ndc80 complex is an elongated complex formed from coiled-coil domains built from the heterodimers Ndc80^Hec1^:Nuf2 and Spc24:Spc25. At the inner end, the RWD domains of Spc24 and Spc25 connect the Ndc80 complex with kinetochores, while the microtubule-interacting CH-domains of Ndc80 and Nuf2 are located at the other end of the rod [23]. There is a break within the coiled-coil domain of Ndc80:Nuf2, the loop region of the Ndc80 complex. It allows the Ndc80 complex to adapt both an elongated, and a bent conformation [24]. This conformational change termed “jackknifing” is linked to both attachment and microtubule dynamics [25]. Intra-kinetochore tension, including the Ndc80 complex conformational change, has been implied to function as a trigger for SAC silencing [13,14,25].

If the physical stretch of either centromere or kinetochore plays an immediate role in attachment stabilization remains an open question. Experimental data suggest an importance of both. Therefore, mechanistic models need to consider both and can only be speculative at this point. The stretch of an individual kinetochore may be required to silence checkpoint signaling at this very kinetochore, e.g., by separating two effector proteins or destabilizing a tension-sensitive recruitment site. Yet, the kinetochore remains susceptible for SAC signals as long as the centromere associated with it remains in a relaxed state. Stretching of the centromere reverts this susceptibility, potentially by spatially separating the kinetochore from effectors situated in the inner centromere. A combination of these two modes of stretching allows for re-probing the attachment state by oscillating the stretches of kinetochore and centromere.

## 3. The players: localizing Mps1 and Ipl1 to the kinetochore

The bulk of mitotic signal integration during EC and SAC signaling is encoded through phosphorylation, therefore the mechanism comes down to a precise interplay between kinases and phosphatases. An important aspect of mechano-sensitive signaling is the balance between phosphorylation and dephosphorylation at kinetochores regulated through the dynamic recruitment of kinases and phosphatases. In general, phosphorylation promotes EC and SAC signaling, whereas dephosphorylation facilitates the progression into anaphase. The two kinases often discussed to be at the signaling apex are Ipl1^Aurora B^ and Mps1 [26,27]. Ipl1 (**I**ncrease in **Pl**oidy 1) was discovered in a genetic screen for genes required for euploidy [28]. Ipl1 defective cells fail to establish bi-orientation and to respond to kinetochore attachments that lack tension [29,30]. Mps1 (**M**ono**P**olar **S**pindle 1) was first identified to be essential for the duplication of spindle pole bodies in budding yeast [31], but it is also crucial for checkpoint activation upon treatment with the microtubule destabilizing drug nocodazole [32]. Both kinases are highly conserved though the phylogenetic tree of life, with only nematodes having lost the Mps1 orthologue [33,34].

Ipl1 is a member of the family of aurora kinases and its only representative in yeast. Most Metazoans express multiple aurora kinases: Aurora A is associated with centrosome duplication and spindle organization [35], Aurora B (the functional orthologue of Ipl1) is associated with chromosome bi-orientation [36,37]. Mammals express a third orthologue, Aurora C, which functions similarly to Aurora B but supports the specific requirements of meiotic divisions in germline cells [38].

**Figure 2.**
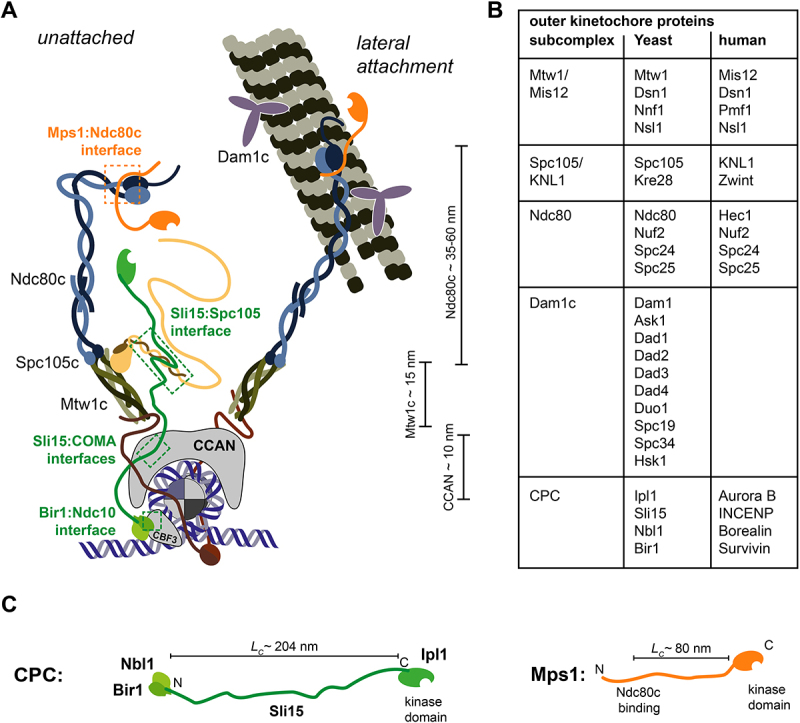
The kinetochore as a scaffold for kinase recruitment. Recruitment mechanisms of Mps1 and Ipl1 (CPC) to the KMN network. A. Illustration highlighting key interfaces for Mps1 at the Ndc80/Nuf2 neck, and for Sli15 at the Spc105/Kre28 helical bundle domain. Note that Mps1 binding to Ndc80c is fully compatible with lateral kinetochore-microtubule attachments. Distances along the kinetochore were determined experimentally by fluorescence microscopy [55]. B. Overview over the outer kinetochore subcomplexes in budding yeast with their subunits and the corresponding counterparts in humans. C. Organization of the CPC and Mps1. The n- and C-termini of Sli15 and Mps1 are indicated. The position of the kinase domain is highlighted. The contour length L_C_ is the maximal possible extension of the protein, assuming all intrinsically disordered segments between kinetochore targeting modules and kinase modules are maximally stretched. It was calculated by multiplying the number disordered residues with 0.38 nm (length of an amino acid backbone) and adding the length of structured segments (single alpha helix for Sli15, SPB targeting domain for Mps1).

Ipl1^Aurora B^ forms the chromosomal passenger complex (CPC) together with Bir1^Survivin^, Nbl1^Borealin^, and Sli15^INCENP^. In the CPC, Ipl1^Aurora B^ functions as the kinase module. To be fully active, it requires interaction with the C-terminal IN-Box of Sli15^INCENP^, as well as trans autophosphorylation of the Ipl1^Aurora B^ T-loop [39,40]. On its N-terminal end, Sli15^INCENP^ forms a three helix bundle with Bir1^Survivin^ and Nbl1^Borealin^. In human and fission yeast cells, Survivin reads a phosphorylation placed on histone H3 Thr3 by Haspin kinase [41–44]. A second interaction is built between Borealin and Shugoshin (Sgo1/2), after Shugoshin is recruited to Bub1-phosphorylated histone H2A Thr120 [45]. In budding yeast, the CPC was found to be recruited to the kinetochore by a different mechanism because (1) neither Sgo1 nor the haspin-like kinases Alk1 and Alk2 are essential [46,47] (2) CPC kinetochore levels are not altered in a *sgo1∆* [48]. A potential receptor is the yeast-specific kinetochore protein Ndc10, which is part of the kinetochore-foundational CBF3 complex. It can interact directly with Bir1 [49,50]. While deletion of Bir1 results in a severe growth phenotype, the lethality can be suppressed by deleting an N-terminal fragment of Sli15 (Sli15^∆N^) which contains the Bir1:Nbl1 binding segment [51]. The Sli15^∆N^ construct remains able to localize with kinetochores as well as spindle microtubules, without co-recruitment of Bir1 or Nbl1 [51]. This observation prompted the search for an alternative recruitment pathway for Ipl1:Sli15 that relied on the kinetochore rather than the centromere. Two interfaces were found to bind Sli15 also in its N-terminal deletion mutant: the COMA subcomplex (situated in the inner kinetochore) through the C-terminal RWD domain in Ctf19 [52,53], and the Spc105 complex (situated in the outer kinetochore) through a conserved helix bundle domain built by both Spc105 and Kre28 [54] (Figure 2A). At this point, it is unclear, whether these kinetochore interfaces are specific to budding yeast. Similar interactions between INCENP and the kinetochore have so far not been reported in human cells, here the recruitment exclusively depends on the centromere targeting by Survivin and Borealin [42,56]. The individual contribution of either interface requires further analysis. The inner kinetochore interactions are the primary driver of CPC kinetochore localization. The Bir1:Nbl1-dependent pathway and the COMA complex pathway act redundantly in this regard [53]. On the other hand, the interaction with the outer kinetochore mediated by the Spc105 complex has only very limited influence on the recruitment of the CPC, but loss of this interaction severely compromises error correction. It remains unclear whether a single CPC engages with multiple recruitment sites simultaneously, or whether distinct CPC pools – recruited via different sites – perform specialized functions.

Recruiting Mps1 to kinetochores is a crucial step of SAC signaling [57]. The protein has a highly conserved kinase domain located C-terminally [58]. Activation of Mps1 requires autophosphorylation of conserved residues within its T-loop [59]. The N-terminus, which is responsible for its localization to kinetochores, is evolutionary divergent. In budding yeast, two independent segments target Mps1 for its role in spindle pole body duplication and SAC signaling [60]. Responsible for SAC signaling is an intrinsically unstructured region (aa110-180), that binds the “neck” of the Ndc80:Nuf2 heterodimer, opposite from the microtubule-binding interface of the Ndc80 CH-domain “head” [61–63]. It is crucial to note that the Ndc80 complex is able to bind Mps1 and microtubules simultaneously [62,64] (Figure 2A). In human cells, the exact mode of binding is less clearly defined. Multiple elements were shown to mediate recruitment: a tetratricopeptide repeat (aa62-192), as well as a middle region, were shown to interact with the Ndc80 complex [65–67]. Based on competition experiments using NMR and solid-phase binding assays, a model was proposed, in which Mps1 competes with microtubules for the interaction with the Ndc80 complex [65,66]. Therefore, Mps1 would only be recruited to unattached kinetochores. The formation of an attachment would then inactivate the SAC by removal of Mps1. This model, however, has been challenged by observations that could show clear Mps1 recruitment to attached kinetochores [68]. An in-depth understanding of the regulated recruitment of Mps1 to the kinetochore requires a comprehensive analysis of the Mps1 binding site, which has not been achieved in human cells yet.

An evolutionary conserved feature to regulate Mps1’s recruitment to kinetochores is autophosphorylation. After co-purification with yeast kinetochores, Mps1 autophosphorylation *in vitro* results in its release from the purified kinetochore [69]. Supporting a conserved role for autophosphorylation in tuning kinetochore affinity, inhibiting Mps1 activity with the small-molecule inhibitor Reversine increases Mps1 levels at kinetochores drastically in human cells [70]. How exactly Mps1 autophosphorylation controls its localization is only poorly understood. Autophosphorylation sites are located within the respective kinetochore targeting domain in both yeast [62] and human [68,71]. In both cases, mutating the phosphorylated residues to alanine results in elevated levels at kinetochores, yet the removal dynamics are not altered in the absence of the autophosphorylation sites. Further research is required to characterize the contribution of Mps1 autophosphorylation in regulating its recruitment.

## 4. The first objective: delay the onset of anaphase

The SAC prevents the transition into anaphase before all chromosomes have achieved bi-orientation. Anaphase is initiated by the activation of the anaphase promoting complex/cyclosome (APC/C) [72]. Before anaphase, the APC/C is inhibited by the mitotic checkpoint complex (MCC), formed from Cdc20, cMad2 (a closed conformation of Mad2), Mad3^BubR1^, and Bub3 [73], which can bind APC/C^Cdc20^ to prevent its access to substrates [74]. The primary function of the mitotic checkpoint is to regulate MCC formation, using kinetochores as catalytic platforms (for a more detailed perspective, we refer the reader to SAC specific reviews [57,75]).

Mps1 sets the foundation for SAC signaling by phosphorylating MELT ([M/I][E/D][I/L/M][S/T]) motifs on Spc105^Knl1^ which allows the recruitment of MCC assembly catalysts [76–78]. Yeast cells overexpressing Mps1 arrest in metaphase while establishing full attachments [79]. The phosphorylated MELT motifs are bound by Bub3, which co-recruits Bub1 [80–83]. Bub1 interacts with Mad1:cMad2 [84,85]. After being responsible for their initial recruitment, Mps1 also acts together with Bub1:Bub3 and Mad1:cMad2, to catalyze the rate-determining step of MCC assembly: the complex formation between cMad2 and Cdc20 [86–91].

Whether Ipl1^Aurora B^ also contributes to SAC establishment directly is debated. Early investigations in budding yeast using the temperature sensitive *ipl1-321* allele revealed a requirement for Ipl1 activity to induce a metaphase arrest in scenarios where kinetochores lack tension (e.g., cohesin mutants or lack of a sister chromatid by preventing replication). However, Ipl1 is not required for arrest induced with the microtubule-depolymerizing drug nocodazole [30]. Together with Ipl1’s role in the establishment of bi-orientation [92], this led to a model where Ipl1 destabilizes tensionless attachments. The resulting unattached kinetochore then activates Mps1-dependent SAC signaling to block anaphase onset. Also, in human cells, inhibition of Aurora B silences the SAC in conditions where kinetochores remain attached without tension but not in nocodazole conditions [36,37]. Thus, unattached kinetochores, not lack of tension *per se*, were considered the primary trigger for SAC signaling, while EC plays an auxiliary role by generating unattached kinetochores from tensionless attachments [93–95].

Distinguishing cleanly between lack of tension and detachment experimentally is challenging because genetic or pharmacological intervention can easily influence both parameters. Treatment of cells with the microtubule-stabilizing drug taxol not only reduces the tension the spindle applies on the centromere, it also leads to the detachment of some kinetochores in human cells. Given that human centromeres are associated with multiple kinetochores, a mixed population within the same centromere is possible in which some kinetochores remain attached, while others are detached and can initiate SAC signaling [11,96]. Budding yeast, on the other hand, has a point centromere, *i.e.,* a single kinetochore module is built per centromere, which interacts with a single microtubule [16]. Reducing tension by taxol treatment in this model system is impeded by the fact that yeast tubulin only interacts with taxol after the introduction of a mutation [97,98]. When such mutants are arrested in metaphase and then treated with taxol, kinetochore microtubule attachments persist, but sister centromeres are no longer separated, suggesting that tension is lost [99]. Taxol treatment results in a delayed anaphase onset after the mitotic arrest was relieved. This progression delay is dependent on Bub1 and Bub3, but not Mad1, Mad2, or Mad3 [99]. This differential requirement suggests that a potential tension-specific SAC activation mechanism may exist alongside the canonical pathway. Assessing the mechanistic details of this putative pathway requires additional research.

Whether the detachment of microtubules is the only contribution of Aurora kinases to SAC signaling, is under debate. Firstly, Ipl1^Aurora B^ is deeply involved in regulating Mps1 recruitment. In budding yeast, Mps1 competes with the C-terminal tail of the outer kinetochore protein Dam1 for a shared binding surface on the Ndc80 complex [62,63]. The interaction between the Ndc80 complex and Dam1 is prevented by Ipl1 phosphorylation (Dam1 Ser257/Ser265/Ser292) [100–102]. These sites need to be dephosphorylated for SAC silencing [62,103]. Yet, the effect on overall Mps1 recruitment seems to be subtle as Ipl1 activity is not strictly required to localize Mps1 at yeast kinetochores as judged by fluorescence microscopy [104]. In human cells, Aurora B activity is required for Mps1 localization [67,68,105]. Recruiting the CPC to anaphase kinetochores is sufficient to recruit Mps1 and Bub1:BubR1, but in a configuration that does not allow for SAC signaling as Mad1:Mad2 are not localized under these conditions [106].

There are also pieces of evidence indicating that Ipl1^Aurora B^ has a more direct involvement in the checkpoint than just regulating Mps1 recruitment. Inhibition of Aurora B has synergistic effects with the loss of SAC signaling from Mps1 or Bub1 inactivation in human cells, suggesting a signaling pathway parallel to the Mps1 dependent one [107–109]. Novel insights into Ipl1^Aurora B^-derived SAC activation were obtained using the “ectopic spindle assembly checkpoint” (eSAC), an artificial rapamycin-inducible dimerization system which tethers a checkpoint kinase (Ipl1 or Mps1) with another component of the checkpoint cascade [110,111]. The eSAC system revealed that Ipl1^Aurora B^ could not arrest cells in metaphase through the Spc105^Knl1^ MELT-motif pathway but was able to do so when tethered to either Bub1 or Mad1 (one needs to note that this pathway could be inactivated by Mps1 inhibition) [111]. The arrest depends on Bub1’s ABBA-motif, which is responsible for an interaction with Cdc20 and required for a functional SAC in human cells [112,113]. This segment was not required for orthogonal experiments tethering Mps1 [111], suggesting a mechanistically distinct Ipl1-dependent pathway. It is possible that this dimerization system reconstituted the tension-specific SAC pathway identified in taxol sensitized yeasts [99]. In both cases, Bub1 plays a crucial role. The contribution of Ipl1 has not been tested in the latter study, but given that tension was a central element, a dependency on Ipl1 is not unlikely. Whether this resembles a conserved pathway and its significance next to the canonical pathway will require further investigation.

## 5. The second objective: correct erroneous attachments

**Figure 3.**
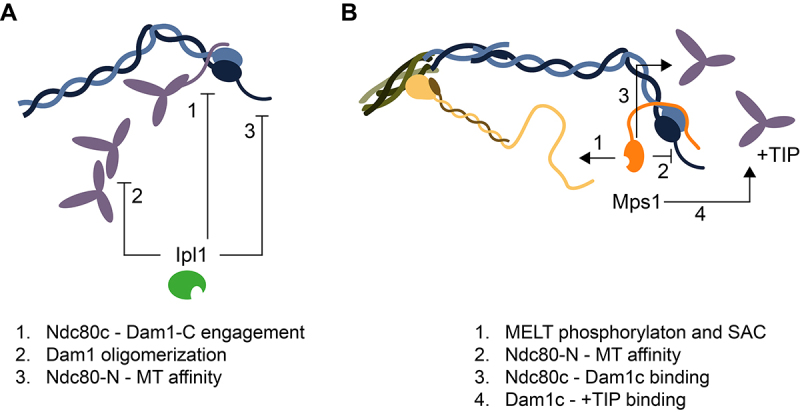
Molecular consequences of Ipl1 and Mps1 kinase activities at the kinetochore. Ipl1 and Mps1-based mechanisms during error correction and SAC. A. Ipl1 activities prevent premature stabilization of attachments by (1) inhibiting the interaction between the Dam1 complex and the Ndc80 complex through phosphorylation of the Dam1 C-terminus, (2) inhibiting the polymerization of Dam1 complex protomers through phosphorylation of the Dam1 N-terminus, and (3) reduction of the affinity of the Ndc80 N-terminal tail towards microtubules B. Mps1 activities promote mitotic fidelity by (1) initiating SAC signaling through phosphorylation of the Spc105 melt motifs, (2) reduction of the affinity of the Ndc80 N-terminal tail towards microtubules, (3) promoting the interaction between the Dam1 complex and the Ndc80 complex through phosphorylations on Ndc80, (4) promoting +tip binding of the Dam1 complex through phosphoregulated interactions with end binding proteins.

While the SAC prolongs metaphase, EC is responsible to destabilize attachments that lack tension and stabilize bi-oriented attachments. The error correction process in budding yeast is very efficient, which may contribute to the fact that the SAC is not essential in this organism. In an unperturbed cell cycle, sister chromatids achieve bi-orientation before the APC/C is activated, and anaphase is initiated via SAC-independent timer mechanisms (e.g., Cdk-dependent APC/C activation [114] or phospho-dependent cleavability of Scc1 [115]). The main player of mitotic error correction is Ipl1^Aurora B^ (Figure 3A). This serine/threonine kinase phosphorylates many substrates within the kinetochore framework. While single phosphorylation sites are often not required individually, Ipl1 conducts their ensemble to switch from attachment stabilization to destabilization.

Tuning the affinity of kinetochores to microtubules is achieved by targeting the microtubule receptors at the outer kinetochore. Microtubule-binding activities of the kinetochore are found in the KMN network (specifically Ndc80, and the N-terminus of Spc105^KNL1^), and in yeasts, the Dam1 complex [19,20,101,116–120]. Given that Ipl1^Aurora B^ destabilizes kinetochore microtubule attachments, the expectation is that phosphorylation reduces the affinity toward microtubules. Work on their vertebrate orthologues revealed that Aurora B phosphorylates the KMN network on all subcomplexes and phosphomimetic aspartate mutants completely disrupt its interaction with microtubules [121]. The Ndc80 complex interacts with microtubules through electrostatic interactions driven by evolutionary conserved lysine residues on the CH-domain of Ndc80 and the adjacent positively charged N-terminal tail [23,119,122]. Ipl1^Aurora B^ targets the Ndc80 N-terminal tail resulting in reduced affinities toward microtubules in yeast and humans [123,124]. The introduced phosphates act by providing negative charges counteracting the electrostatic interactions between positively charged residues on Ndc80 with the negatively charged tails of tubulin [122]. The Ipl1 consensus with a preferred K/R at the −2 position is ideally suited to tune microtubule affinities in this manner [125]. Spc105’s interaction with microtubules is mediated by a basic patch positioned at its N-terminal end [126]. Aurora B targets a serine residue within the binding interface, reducing the affinity for microtubules significantly [121]. Although the Mis12 complex does not directly interact with microtubules, mutations mimicking Aurora B phosphorylation on the Mis12 complex potentiate the effect of mutants in the other KMN members that drive the microtubule interaction [121].

The fungal-specific Dam1 complex is a heterodecamer which further oligomerizes into rings around microtubules [127]. Ipl1 phosphorylates the subunits Ask1, Dam1, and Spc34. Phosphorylation mutants show pronounced chromosome missegregation [100]. Phospho-null mutants aggravate the segregation defects of *ipl1* mutants, while phospho-mimetic mutations suppress them, arguing that Dam1c constitutes a key target. In difference to the phosphorylation on the KMN network, the phosphorylation sites on the Dam1 complex are not situated at the interface with microtubules. Instead, they modulate the interactions within the outer kinetochore. The phosphorylated residues on Dam1’s C-terminus (Ser257/ Ser265/ Ser292), Ask1 (Ser200), and Spc34 (Ser199) are located in the interface between the Dam1 complex and the Ndc80 complex [102]. These interfaces are disrupted upon Ipl1 phosphorylation resulting in a destabilized attachment [119,128]. On top, Ipl1 phosphorylates Ser20 in Dam1’s N-terminus, which forms a “staple” between adjacent Dam1 complex decamers upon ring formation [102]. When phosphorylated by Ipl1, the cooperativity of Dam1 complex decamers is lost, and the overall affinity is decreased [129]. This indicates that Ipl1 controls Dam1 complex oligomerization via phosphorylation of Dam1 Ser20. Measurements of forces *in vitro* indicate a special importance of this mechanism to withstand higher forces [130]. As long as Ipl1 activity is high, the outer kinetochore is prevented from “locking” prematurely into a Dam1-dependent end-on attached state. Together, these Ipl1^Aurora B^ dependent modifications provide a basis to destabilize erroneous attachments.

Besides Ipl1, Mps1 has also been identified to contribute to error correction (Figure 3B). In yeast, studying Mps1’s contribution to error correction was complicated, as Mps1-deficient cells cannot duplicate their spindle pole bodies [31]. These cells have monopolar spindles and cannot form bi-oriented attachments by definition. Approaches combining chemical genetics with temperature-sensitive alleles could identify a dependence on Mps1 activity for the establishment of bi-orientation after the spindle pole body was duplicated [104,131]. Further, the identification of separation-of-function alleles of Mps1, which disrupted kinetochore associated functions while retaining the duplication of spindle pole bodies, revealed that the loss of Mps1 at kinetochores results in more severe growth and mis-segregation phenotypes than a SAC deletion [61,62]. The precise mechanism of Mps1’s contribution to error correction is only poorly understood. In higher eukaryotes which have both Aurora A and B kinases, Mps1 enhances the activity of Aurora A kinase which leads to the destabilization of microtubule kinetochore attachments through phosphorylation of the Ndc80c [132]. Because Aurora A is enriched at the spindle poles, this allows Mps1 to indirectly detach chromosomes, which are not properly positioned at the metaphase plate. Given that yeasts do not have Aurora A, Mps1 must contribute to EC in additional ways as well. When co-purified with native kinetochores from yeast lysates, Mps1 activity can destabilize the interaction between the kinetochores and microtubules. To do this, it targets serine and threonine residues within the N-terminal tail of Ndc80 [133]. However, if Mps1 activity also results in detachment *in vivo* is unclear. Upon overexpression of Mps1, budding yeast cells become arrested in metaphase with attached kinetochore [79]. Indeed, Mps1 has also been associated with mechanisms one would expect to promote attachment stabilization. In human cells, Mps1 is required for multiple steps of the expansion of the fibrous corona around unattached kinetochores [134]. Besides its role in SAC signaling, the kinetochore corona also contributes to the attaching the kinetochore by inducing microtubule nucleation [135]. Most components of the kinetochore corona are not conserved between human and yeast but the proteins Stu1 and Slk19 could present functional orthologues. The two proteins accumulate at unattached kinetochores via phosphorylated MELT motifs in Spc105, potentially as a Stu1:Slk19 polymer which supports the clustering of unattached kinetochores in an Mps1-dependent manner [136,137]. The microtubule polymerization function of Stu1 supports the capture of unattached chromosomes [138,139].

In budding yeast, Mps1 activity at kinetochores is required for stable attachments of the Dam1 complex [61]. This stabilization is driven by the phosphorylation of Dam1 on Ser218 and Ser221 by Mps1 [140], as well as by the phosphorylation of a helical segment of Ndc80 [141]. Mutating the phosphorylation sites in Dam1 to alanine prevents the formation of stable end-on attachments and results in meiotic but not mitotic, chromosome segregation defects [140,142,143]. Additionally, Mps1 activity also promotes the association of Dam1 complex with the +-end-binding protein Bim1 [144]. While co-immunoprecipitation experiments show that the stable kinetochore integration of Dam1 complex depends on Mps1 activity [61], this does not result in a reduction of co-localization when analyzed by fluorescence microscopy [62,140]. Loading Dam1 on kinetochores thus appears to be a multi-level process in which some steps require Mps1 activity. In conclusion, while strong evidence suggests key contributions by Mps1 in regulating kinetochore attachments, the exact mechanisms, if they have a stabilizing or a destabilizing effect, and if they act sequentially or in parallel, remain to be investigated.

## 6. The synopsis: connecting kinetochore stretching to the modulation of Ipl1 and Mps1 activities

Stretching of centromeres and/or kinetochores needs to be translated into biochemical activities. The predominant model for CPC regulation is the spatial separation model, derived from experiments in human cells using FRET sensors for Aurora B activity. These sensors were tethered either to the centromere (fused to CenpB) or to the outer kinetochore (fused to Mis12) [145]. While the centromere-linked sensor was unaffected by perturbations of tension, the phosphorylation of the kinetochore-positioned sensor was reduced under circumstances that place centromeres under tension. A complementary experiment was performed in budding yeast, where the attachment status of one centromere was followed upon inducible tethering of the CPC [146]. If the CPC was tethered to the outer kinetochore (via the Ndc80 C-terminus), cells lost bi-oriented attachments and failed to newly establish them. Tethering the CPC to the inner kinetochore (via the Mif2^CenpC^ C-terminus) did not impede EC. The dependence of correct CPC function on its spatial relation to its targets is therefore a conserved feature from yeast to humans.

The mechanistic understanding depends on the understanding of localization mechanisms of the CPC. The human CPC has a well-defined centromere targeting module formed from Survivin : Borealin : INCENP^N-terminus^, which binds centromeric chromatin. Aurora B is positioned on INCENP’s C-terminus, using INCENP as a flexible linker. When tension stretches the centromere, the outer kinetochore is physically separated from the CPC’s anchor point. In budding yeast, annotating these structural components requires further dissection. Recruitment to the inner-kinetochore can occur through two redundant processes: Bir1’s interacts with the CBF3 complex and Sli15’s interaction with the COMA complex. While neither the centromere targeting module nor the Mcm21:Cf19 subunits are essential, they show synthetic lethality [52,53]. The spatial separation model could explain CPC function based on only tethering the inner kinetochore module. Nevertheless, the outer kinetochore Spc105:Kre28 complex was identified to interact with Sli15. From the finding that this interface is essential even when disrupted in a wildtype Sli15 [54], one can infer that the essential CPC function not only requires the outer kinetochore to be proximal enough for Ipl1^Aurora B^ to reach it, but the outer kinetochore must also provide a binding interface. The reason for this still remains to be investigated. Possible hypotheses are that a binding event might increase the time the CPC’s kinase module resides at the outer kinetochore, or the interaction between Sli15 and the KMN network locks either of the partner in a conformation which supports error correction. Recent studies have investigated the structural coordination of the KMN network, both in human and yeast proteins. Whereas the coiled-coil domains formed by Knl1:Zwint in human cells are positioned stiffly parallel to the coiled-coil domains of Spc24:Spc25 [147,148], such a structure could not be resolved for their yeast homologues [149]. The RWD domains, contributed from both Spc105 and Kre28, coordinate their recruitment to the Mis12 complex. The coiled-coil domains in Spc105 and Kre28 are connected via two flexible linkers, allowing to speculate for a conformational re-orientation of the Sli15 binding site located in the coiled-coils [54]. If the re-orientation was guided by the microtubule-binding site located in Spc105’s extreme N-terminus, this model would also connect Ipl1 activity to attachment and/or tension.

Conformational changes in the outer kinetochore also modulate the activity of Mps1. If Mps1 is stably tethered to kinetochores, the tether’s position is a determinant for the induction of a metaphase arrest in budding yeast [150]. Physiologically, Mps1 is recruited to the CH-domains of the Ndc80 complex, the outmost element of the kinetochore. Stable recruitment of Mps1 to this position or to subunits of the Dam1 complex still allows for cellular viability, whereas positioning of Mps1 further toward the inner kinetochore, even on the centromere-proximal side of Ndc80, results in metaphase arrest and loss of viability [150]. The lethality is the consequence of an overactivation of the SAC, as it depends on the MELT motifs within Spc105. Recruiting Mps1 to the Ndc80 complex CH-domains may allow for an attachment-dependent separation of Mps1 from Spc105, explained by the “jackknife”-model for Ndc80 complex’s structure [25]. This implies direct contributions from kinetochore stretching on SAC silencing by separating Mps1 from its targets without stripping it from kinetochores. Mps1 removal is achieved independently by a competition mechanism between Mps1 and the C-terminus of Dam1. The balance between Mps1 and Dam1 for binding the Ndc80 interface is controlled by Ipl1 phosphorylation on Dam1’s C-terminus (Ser257/ Ser265/ Ser292) [62,119]. Phospho-regulating the recruitment to this binding hub on the Ndc80 complex neck marks a conserved mechanism as the autophosphorylated residues in Mps1, which impair its kinetochore recruitment, structurally superpose their Ipl1 phosphorylated counterparts in Dam1 [63]. Concluding, Mps1 activity at kinetochores is controlled by kinetochore stretching on two levels in budding yeast. Stretching the bent conformation of the Ndc80 complex separates it from Spc105, separating the CPC from the Dam1 complex allows for the removal of phosphorylation on Dam1’s C-terminus resulting in the displacement of Mps1 from the kinetochore.

The onset of anaphase is associated with a transition from a net phosphorylation state, dominated by mitotic kinases, toward a net dephosphorylation state in which phosphatases take over. At the kinetochore, this requires not only the removal of mitotic kinases, it also needs to favor the recruitment of phosphatases. The kinetochore can recruit protein phosphatase 1 (Glc7^PP1^) via evolutionary conserved [S/G]ILK and RVSF motifs, situated in the N-terminus of Spc105^KNL1^ [151,152]. These docking sites are subject of regulation by Ipl1^Aurora B^. Phosphorylation of proximal serine and threonine residues reduce the binding between Spc105^KNL1^ and Glc7^PP1^ [151]. Once a mature attachment is established, Ipl1^Aurora B^ cannot phosphorylate Spc105^KNL1^, allowing for full recruitment of Glc7^PP1^. Kinetochore-bound Glc7^PP1^ is required for the silencing of the mitotic checkpoint. It dephosphorylates the MELT-motifs in Spc105^KNL1^ [78,153]. In Drosophila, where the MELT motifs in Spc105 are lost [154,155] contributes to SAC silencing by removal of an activating phosphate in Mps1’s T-loop [156]. In budding yeast, Glc7 activity limits Mps1’s ability to bind the kinetochore by dephosphorylating the Dam1 C-terminus, thereby making it a potent competitor for the neck region of the Ndc80 complex [100]. Glc7^PP1^ not only silences the checkpoint acutely, it also prevents reactivation of the checkpoint in anaphase.

Another mitotically important phosphatase is protein phosphatase 2A (PP2A). PP2A is a heterotrimer, built from a catalytic subunit (PP2A-C), a scaffold subunit (PP2A-A), and a substrate determining regulatory adapter subunit (PP2A-B). In human cells, special emphasis is put on the PP2A:B56 complex. It is recruited to the kinetochore via a direct interaction with BubR1 [157], which is recruited to phosphorylated MELT motifs in KNL1 using Bub3 as an adapter [158]. Once recruited, PP2A:B56 dephosphorylates the MELT motifs, providing a negative feedback-loop as Mps1 activity results in the recruitment of its opponent [159]. Besides counteracting Mps1 in regards to MELT motif phosphorylation, PP2A:B56 also limits its recruitment by counteracting Aurora B phosphorylation (at an unknown site) which is crucial for Mps1 recruitment in humans [68]. In budding yeast, PP2A is recruited in complex with the adapter protein Rts1 which build a conserved interaction with Sgo1 [160–162]. While deletion of Rts1 delays both mitotic progression and the removal of pericentromeric Sgo1, disrupting the interaction between Sgo1 and Rts1 does only the latter [163]. Therefore, PP2A:Rts1 might contribute to a timely mitotic progression in a Sgo1-independent pathway.

## 7. Development of a perspective

**Figure 4.**
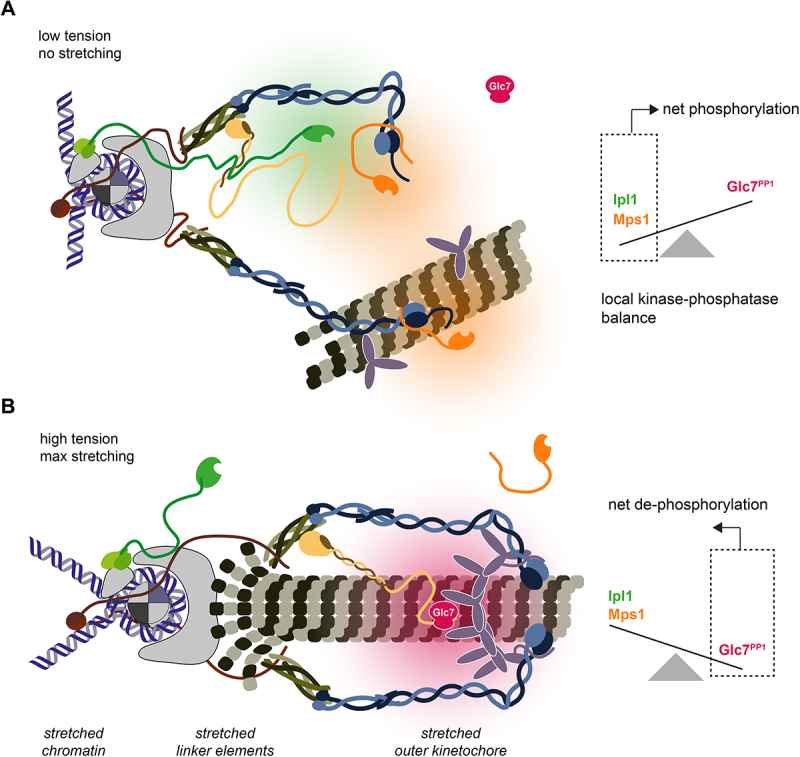
Structural transitions of the kinetochore controlling Ipl1 and Mps1 activities. A. A kinetochore-lacking tension provides recruitment surfaces for Mps1 and Ipl1 in close proximity to their substrates. As part of the CPC, Ipl1 recruited via interactions with centromeric chromatin, subunits of the CCAN, and the Spc105:Kre28 complex of the outer kinetochore. Mps1 is recruited to the neck region of the Ndc80 complex. The high local concentration of kinases favors phosphorylation over de-phosphorylation. B. Upon exertion of tension, the kinetochore is stretched and the distance between inner and outer kinetochore increases. In this configuration, the centromere bound CPC cannot reach its interaction partner at the outer kinetochore. Ipl1 phosphorylation is reversed by the Glc7^PP1^ phosphatase. The Dam1 complex oligomerizes and the Dam1 C-terminus replaces Mps1 at the Ndc80 complex neck.

Understanding how the biophysical concept of tension-establishment is translated into the two biochemical mechanisms error correction and the spindle assembly checkpoint is one of the main open question in mitotic research. Based on the evidence presented, we propose a model for the maturation of kinetochore microtubule attachments in budding yeast (Figure 4A,B). Kinetochores are unattached after their assembly. Mps1 is recruited through the Ndc80 complex and phosphorylates MELT motifs on Spc105 to initiate SAC signaling and promote chromosome capture through Stu1. Initial contacts with microtubules are made by lateral attachments. Mps1 activity promotes the interaction between the Ndc80 complex and the Dam1 complex to facilitate end coupling, thereby supporting the lateral-to-end conversion of the attachment. Whether the end-on attachment is stabilized or destabilized depends on the level of Ipl1 activity at the outer kinetochore, which is determined by the occurrence of kinetochore stretching as a consequence of tension. If the attachment cannot generate tension, Ipl1 phosphorylates the Dam1 complex, preventing a tight interaction with the Ndc80 complex as well as ring formation. Ipl1 and Mps1 phosphorylation on the N-terminal tail of Ndc80 result in the release of the microtubule from the kinetochore. This allows for a new attachment to be formed and supports SAC signaling in the meantime. If the attachment can generate tension, Ipl1 is spatially separated from its substrates at the outer kinetochore and Mps1 is spatially separated from Spc105. Glc7^PP1^ removes phosphates previously placed by Ipl1 and Mps1. The Dam1 complex builds tight interactions with the Ndc80 complex and forms oligomers around the microtubule. The interaction between the Ndc80 complex and the C-terminus of Dam1 allows for a robust coupling of kinetochores and microtubules and ensures the delocalization of Mps1 from kinetochores, preventing a reactivation of SAC signaling in anaphase when tension drops. The SAC is silenced and the cell proceeds into anaphase. The activity and regulation of Mps1 represents a negative feedback loop as Mps1 supports the interaction between the Ndc80 complex and the Dam1 complex, thereby promoting the recruitment of its own competitor. The tension-dependent regulation of Ipl1 activity serves as a regulatory checkpoint in the feedback loop at kinetochores that have not achieved bi-orientation.

Key questions concerning the molecular basis of mitotic fidelity remain unanswered. One main challenge is the lack of understanding how a kinetochore is constructed *in situ* and how end-on attachment and the generation of tension remodel its structure. Improvements in electron microscopy have advanced toward a structural perspective on reconstituted kinetochore complexes [17,18,102,147,149]. Tomographic reconstruction of purified chromosomes could identify proteinaceous densities corresponding to kinetochores [164]. Technical limitations have so far prevented to resolve subcomplexes of the outer kinetochore and microtubules were not part of the analyzed specimen. Understanding how kinetochores are embedded within chromosomes on the one side and the mitotic spindle on the other, requires to analyze their organization also in the cellular context. To this end, light microscopy-based approaches were applied to determine kinetochore composition and, as far as possible, structure. Albeit the limitations of light microscopy are partially overcome through techniques such as super-resolution microscopy or computational tools [9], higher resolution data using electron microscopy/tomography will be required to determine how kinetochore subcomplexes react to the establishment of bi-orientation.

Also, the molecular perspective on error correction does not yield a complete picture yet. Mps1 plays crucial, yet seemingly conflicting roles: promoting *and* impeding attachment stability. Key questions are as follows: Are both roles physiologically relevant? If so, does Mps1 perform them simultaneously or sequentially? The relevant targets need to be identified to dissect the pathways at play. Analyzing their role individually is challenging given the notion that disrupting the Mps1 function in kinetochores will not only influence error correction but also disable the SAC. The conserved nature of the inhibitory effect of Mps1 autophosphorylation on its kinetochore localization implies functional importance. Why was the regulated mechanism evolutionarily favored over an unregulated form of reduced affinity? Is a counterplay between Mps1 and a specific phosphatase required to finetune Mps1 activity in either a spatial or temporal manner? The role of mitotic phosphatases has been widely omitted in this article, as we focused on the regulation of kinases. Phosphatases are of similar importance to the balance and their activity at kinetochore changes during attachment maturation. How is the regulation linked on the molecular level? Is there a contribution by spatial rearrangement of the kinetochore? Further research is required to unravel the full picture of mitotic signaling.
